# Therapeutic Opportunities for Food Supplements in Neurodegenerative Diseases

**DOI:** 10.3390/nu17091527

**Published:** 2025-04-30

**Authors:** Andrea D. Praticò

**Affiliations:** Unit of Pediatrics, Department of Medicine and Surgery, University of Enna Kore, 94100 Enna, Italy; andrea.pratico@unikore.it; Tel.: +39-0935536111

## 1. Introduction

Neurodegenerative diseases (NDs) constitute a group of debilitating conditions characterized by the progressive loss of the structure and function of neurons in the central nervous system. Among the most prevalent are Alzheimer’s disease (AD), Parkinson’s disease (PD), Huntington’s disease (HD), and amyotrophic lateral sclerosis (ALS), as well as other These disorders share common pathogenic mechanisms such as oxidative stress, neuroinflammation, mitochondrial dysfunction, protein aggregation, and synaptic loss, which ultimately lead to cognitive and motor decline [1,2]. With aging populations worldwide, the prevalence of NDs is increasing at an alarming rate, imposing a growing burden on healthcare systems, caregivers, and society at large [3]. Despite decades of intense research, current therapeutic strategies are primarily symptomatic, and no curative treatments are yet available [4].

In this challenging landscape, the scientific and medical communities are increasingly turning their attention to preventive and adjunctive strategies that may slow disease progression, reduce symptom severity, or improve quality of life. Among these, nutritional interventions and dietary supplementation have emerged as promising, low-risk options. A growing body of preclinical and clinical evidence suggests that specific nutrients, phytochemicals, and food-derived compounds may exert neuroprotective effects through diverse biological mechanisms. These include antioxidant and anti-inflammatory actions, enhancement of mitochondrial function, promotion of autophagy, modulation of neurotransmitter systems, and preservation of the blood–brain barrier [5,6,7]. Furthermore, the gut–brain axis—an area of burgeoning interest—has revealed how diet and microbiota composition can influence brain function and inflammation, suggesting a new frontier for dietary modulation of neurological health [8].

Food supplements such as omega-3 fatty acids, polyphenols (e.g., curcumin, resveratrol), vitamins (e.g., D, B12), probiotics, and plant-derived compounds have demonstrated encouraging results in models of neurodegeneration. Some clinical trials have reported cognitive benefits or disease-modifying potential, though replication and large-scale validation remain necessary [9]. The relative safety, accessibility, and cost-effectiveness of food supplements also make them attractive candidates for early intervention or long-term adjunctive care in neurodegenerative diseases. Importantly, these approaches may also hold value in pre-symptomatic or at-risk populations, potentially contributing to primary prevention strategies [10].

This Special Issue of *Nutrients* titled “Therapeutic Opportunities for Food Supplements in Neurodegenerative Disease” was curated to provide a comprehensive platform for exploring these emerging avenues. It brings together original research, systematic reviews, and clinical investigations that highlight the mechanistic underpinnings, therapeutic applications, and translational potential of dietary supplements in the context of NDs. These contributions collectively advance our understanding of how nutrition can influence the neurodegenerative cascade, and they offer a foundation for integrating nutritional science more fully into neurological research and care.

This Special Issue is particularly timely given the current emphasis on integrative and personalized medicine. As interest grows in holistic and lifestyle-based approaches to health, the inclusion of evidence-based dietary strategies could represent a meaningful addition to traditional pharmacological treatments. The articles collected herein demonstrate the multidimensional role of food supplements—from molecular modulation of neuroinflammation and oxidative stress to behavioral outcomes in animal and human studies. By embracing the complexity of neurodegenerative disease biology, the contributions in this issue reflect the importance of multidomain interventions and signal new directions for future research and clinical practice.

## 2. Omega-3 Fatty Acids and Gut Microbiota Modulation

A pilot study by Altendorfer et al. investigates the impact of eicosapentaenoic acid (EPA), an omega-3 polyunsaturated fatty acid, on gut microbiota composition and neuroinflammation in aged wild-type and APP/PS1 Alzheimer’s mice. The study demonstrates that short-term EPA supplementation alters gut microbiota by increasing butyrate-producing bacteria (Firmicutes) and decreasing lipopolysaccharide-producing bacteria (Bacteroidetes). Additionally, EPA reduces the expression of major histocompatibility complex class II (MHCII) in the hippocampus and retina, suggesting a decrease in neuroinflammatory markers. These findings highlight the potential of EPA in modulating the gut–brain axis and attenuating neuroinflammation associated with AD.

## 3. Vitamin D Supplementation and Early Childhood Development

Praticò et al. conduct a retrospective study examining the effects of varying durations of vitamin D supplementation on developmental milestones in early childhood. Comparing cohorts receiving supplementation for 6 versus 12 months, the study finds that extended supplementation is associated with earlier achievement of milestones such as walking and speech. While acknowledging potential socio-environmental influences, the results suggest that prolonged vitamin D intake may positively influence neuromuscular and cognitive development, underscoring the importance of adequate nutritional support during critical developmental periods.

## 4. Polyphenols in Neuroprotection

The neuroprotective properties of polyphenols are further explored in studies focusing on specific compounds:

- Ellagic Acid in Parkinson’s Disease: Radwan et al. investigate the effects of ellagic acid, a polyphenol found in various fruits and nuts, in a mouse model of PD. The study demonstrates that ellagic acid reduces the spread of α-synuclein aggregates and enhances autophagic flux, leading to the preservation of dopaminergic neurons. These findings suggest that elagic acid may mitigate neurodegeneration in PD by promoting the clearance of toxic protein aggregates.
- Astaxanthin and Spirulina Combination: Martin et al. assess the neuroprotective effects of astaxanthin microencapsulated with spirulina in a rat model of lipopolysaccharide (LPS)-induced cognitive impairment. The combination treatment prevents cognitive deficits and reduces microglial activation in the hippocampus and prefrontal cortex. Additionally, it partially reverses LPS-induced gut dysbiosis, indicating a multifaceted approach to neuroprotection involving anti-inflammatory and gut-modulating properties.

## 5. Probiotics and the Gut–Brain Axis

The role of probiotics in modulating the gut–brain axis is examined in a systematic review by Reiriz et al. focusing on the administration of Bifidobacterium infantis and Bifidobacterium breve in neurodegenerative diseases. The review compiles evidence suggesting that these probiotic strains exert neuroprotective effects, potentially delaying disease progression in conditions like AD and PD. The authors highlight the need for further clinical trials to substantiate these findings and elucidate the underlying mechanisms.

## 6. Dietary Diversity and Neurodevelopment in Toddlers

Cunha-Rodrigues et al. conduct a cross-sectional study assessing the association between dietary diversity, food processing, and neurodevelopment in toddlers. The study finds that higher consumption of unprocessed or minimally processed foods correlates with better neurodevelopmental outcomes, particularly in female toddlers. These results emphasize the importance of dietary quality in early life and its potential long-term impact on cognitive development.

## 7. Honey as a Neuroprotective Agent

A review by Fadzil et al. explores the potential use of honey as a neuroprotective agent in managing neurodegenerative diseases. The review compiles in vitro and in vivo studies demonstrating honey’s antioxidant, anti-inflammatory, and anticholinesterase activities, attributing these effects to its high polyphenol content. The authors call for more clinical interventions to validate honey’s efficacy in neuroprotection and its possible integration into therapeutic strategies for NDs.

## 8. Genotype–Phenotype Correlation in Phenylketonuria

Consentino et al. present a study on the genotype–phenotype correlation in a cohort of eastern Sicilian patients with phenylketonuria (PKU). The research underscores the importance of early diagnosis through newborn screening and highlights the variability in phenotypic expression despite similar genotypes and response to dietary norms (low phenylaniline intake) and to newer drugs (i.e., Kvan). The findings advocate for personalized dietary management and continuous monitoring to optimize patient outcomes.

## 9. Natural Compounds with Neuroprotective and Antidepressant Potential

Another significant contribution in this Special Issue explores the neuroprotective and antidepressant properties of Polygonati Rhizoma (PR), a traditional medicinal food with recognized pharmacological activity. In their study, Wei et al. combined systematic pharmacology, molecular docking, and in vitro experiments to investigate the bioactive compounds in PR and their mechanisms of action in a model of depression. Among the identified components, DFV (diosgenin ferulate variant) showed promising binding affinity with COX2 and exerted strong anti-inflammatory and antioxidant effects in LPS-induced BV-2 microglial cells. Notably, DFV reversed mitochondrial dysfunction, inhibited pro-inflammatory cytokines (IL-1β, TNF-α, IL-6), and suppressed the activation of the NLRP3 inflammasome and caspase-1—key regulators of neuroinflammation. These findings suggest that PR may offer a novel, food-derived therapeutic approach to depression and potentially other neuroinflammatory conditions. This study adds to the growing evidence supporting the integration of food-based compounds in managing mood and neurodegenerative disorders.

Collectively, the articles in this Special Issue provide compelling evidence supporting the therapeutic potential of various dietary supplements in neurodegenerative diseases, consistent with findings from other studies involving both primary neurological disorders and diseases associated with neurological involvement [1,2,3,4,5,11,12,13,14]. They underscore the importance of nutritional interventions in modulating disease pathways, promoting neuroprotection, and enhancing cognitive function. While these findings are promising, further large-scale clinical trials are essential to establish definitive efficacy, optimal dosages, and long-term safety profiles. Future research should also aim to elucidate the precise mechanisms through which these dietary components exert their neuroprotective effects, facilitating their integration into comprehensive treatment strategies for neurodegenerative diseases.

## Data Availability

The data supporting the study can be requested from the corresponding Author.
